# Beneficial Effects of Narcotism in Some Obstinate Cases of Neuralgia

**Published:** 1842-03

**Authors:** 


*Beneficial Effects of Narcotism in some obstinate cases of Neuralgia.*—M. Levrat read to the Academy of Medicine at their meeting of the 8th of June last, a memoir on this subject. From the results of his long practice, it appears that opium given to the extent of producing narcotism, often cures cases of neuralgia which have resisted the most varied and most active treatment. This mode of treatment may appear alarming at first, but when directed by a physician who can watch the progress of the phenomena, regulate the administration of the drug, and combat its effects when excessive, it is not dangerous. Five cases successfully treated by this method were related.

